# Physical realization of the Glauber quantum oscillator

**DOI:** 10.1038/srep15816

**Published:** 2015-11-02

**Authors:** Silvia Gentilini, Maria Chiara Braidotti, Giulia Marcucci, Eugenio DelRe, Claudio Conti

**Affiliations:** 1 Institute for Complex Systems, National Research Council, Via dei Taurini 19, 00185 Rome (IT); 2 Department of Physics, University Sapienza, Piazzale Aldo Moro 5, 00185 Rome (IT); 3 Department of Physical and Chemical Sciences, University of L’Aquila, Via Vetoio 10, I-67010 L’Aquila (IT)

## Abstract

More than thirty years ago Glauber suggested that the link between the reversible microscopic and the irreversible macroscopic world can be formulated in physical terms through an inverted harmonic oscillator describing quantum amplifiers. Further theoretical studies have shown that the paradigm for irreversibility is indeed the reversed harmonic oscillator. As outlined by Glauber, providing experimental evidence of these idealized physical systems could open the way to a variety of fundamental studies, for example to simulate irreversible quantum dynamics and explain the arrow of time. However, supporting experimental evidence of reversed quantized oscillators is lacking. We report the direct observation of exploding *n* = 0 and *n* = 2 discrete states and Γ_0_ and Γ_2_ quantized decay rates of a reversed harmonic oscillator generated by an optical photothermal nonlinearity. Our results give experimental validation to the main prediction of irreversible quantum mechanics, that is, the existence of states with quantized decay rates. Our results also provide a novel perspective to optical shock-waves, potentially useful for applications as lasers, optical amplifiers, white-light and X-ray generation.

One of the fundamental problems in physics is the description of irreversible processes, and in particular of their most representative example: an observable physical quantity that decays exponentially. The problem orginates from the fact that exponential dynamics can be excluded from first principles in leading theoretical models, such as the Hilbert space (HS) formulation of quantum mechanics (QM)^1^,^2^, and Hamiltonian classical mechanics^3^,^4^. Exponential dynamics is often explained by a phenomenological coupling^5^,^6^ with enviroment; but in many cases this coupling is negligible, or so small that it cannot quantitatively explain the observed decay rate. A simple model that explains exponential dynamics without coupling with the environment has been theoretically discussed by Glauber^7^, and is based on the harmonic oscillator (HO) paradigm.

As illustrated in Fig. 1, a system with an arbitrary energy potential *V* will behave as a HO in proximity to a local minimum of *V*. The one-dimensional (1D) HO Hamiltonian for the position momentum operators 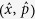 reads as:


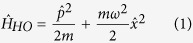


where *m* is the particle mass and *ω* is the angular frequency of the corresponding classical system. 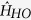 exhibits discrete energy levels 
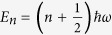
, where *n* = 0, 1, 2.. and *ħ* is the reduced Plank constant. Physical models based on the HO obey time-reversal-symmetry and rule out phenomena such as the origin of the Universe, wavefunction collapse, and the spontaneous decay of atoms and nuclei. However, in his original work^7^, Glauber considers a HO with a negative potential and kinetic energy, and shows that such a system is capable of describing amplification and exponential damping at the quantum level. Glauber, however, points out that quantum inverted oscillators are not avaible “off the shelf” and may seem a bit unrealisitic, as they could, in his words, even solve the world’s energy problems. In turn, the leading feature of the Glauber oscillator is the existence of an infinite number of states with negative energy. While these cannot be achieved in practice, it is possible to conceive systems that do have a reversed potential energy while the kinetic energy remains positive. These latter systems are known as the “reversed oscillators” (RO)^4^,^5^,^8^,^9^. In the RO the levels with negative energy of the original Glauber models are mapped to states with discrete decays rates. At any negative quantized energy corresponds a state with quantized decay rate.

As shown in Fig. 1, at variance with the HO, 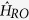 corresponds to a system in proximity to a maximum in its potential energy *V* so that


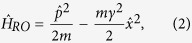


where *γ* is the decay rate of the corresponding classical system. The eigenstates of the RO decay exponentially with discrete decay rates Γ_*n*_/2 = (*n* + 1/2)*ħγ*^9^ and correspond to the so-called Gamow vectors (GVs), originally introduced in nuclear physics^10^. More recently, GVs have been used to describe the arrow of time, and the beginning of the Universe^11^.

In standard Hamiltonian many-body systems, time-reversal is always theoretically possible and the fact that it cannot be observed is relegated to the practical impossibility of inverting dynamics with sufficient precision in an accessible time. In turn, the reversed oscillator (RO) may be identified as the paradigm for irreversible systems where no time-symmetry holds. A complete theory of nature, including both conservative and dissipative processes, should also involve the RO paradigm. Despite an overwhelming amount of data testifying the existence of HO and their quantized energy levels, no one has ever reported evidence of a quantized RO with its signature discretization of decay rates. Hence, the detection of quantized decay rates represents a landmark for the development of a complete theory of nature, shedding light onto phenomena such as the origin of the universe, the irreversibility of quantum measurement, and the spontaneous decay of atoms and nuclei.

As a specific example of a system encompassing an irreversible dynamics, we consider the generation of spatial dispersive shock waves (DSW) during nonlinear optical propagation. DSW occur during highly nonlinear beam evolution in a defocusing nonlinear medium and correspond to the generation of a steep wavefront accompanied by fast oscillations. The fact that DSW are a reversible process is still largely debated in the literature^12^,^13^,^14^,^15^,^16^. In this manuscript, we show that Gamow vectors are excited during this classical wave-breaking phenomena. This fact allows us to report the first experimental evidence of the quantization of Gamow vector decay rates, which demonstrates the physical realization of the Glauber’s inverted oscillator.

## Nonlocal nonlinear optics and the reversed oscillator

We show in the following how a RO occurs in nonlocal nonlinear optical dynamics, which can hence be taken as an analog for simulating irreversible quantum mechanics and as a classical system encompassing GVs. The full details of the theoretical analysis can be found in^17^. In nonlocal media, the onset of wave-breaking phenomena has been reported (see^12^ and references therein); however the analysis is limited to the hydrodynamic approximation of the NLS that only predicts the position of the shock point. GVs allow a complete treatment of all shock wave dynamics.

We start from the paraxial wave equation for the propagation of an optical beam with amplitude *A* and wavelength *λ* in a medium with refractive index *n*_0_, and negligible linear loss (*k* = 2*πn*_0_/*λ*)^12^


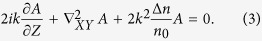


In (3) Δ*n* is the nonlocal refractive index perturbation which can be calculated in terms of the material response function *R* and of the optical intensity |*A*|^2^ by a convolution integral (denoted by an asterisk) Δ*n* = R*|*A*|^2^ 12.

In the one-dimensional case, for a defocusing nonlinearity (*n*_2_ < 0), we write Eq. (3) in terms of dimensionless variables, letting *W*_0_ be the beam waist and *P* the beam power we have:


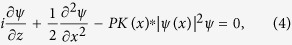


In the highly nonlocal approximation^18^, we have


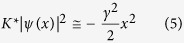


where *γ* is the coefficient of the parabolic approximation of the kernel function. Eq. (4) is formally identical to a quantum system with Hamiltonian (2)


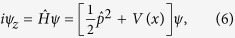


with 
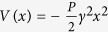
 and 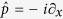.

This argument shows that the nonlinear propagation in a nonlocal defocusing medium leads in a straigthforward way to a model formally equivalent to a RO. For the RO, GVs are numerable and non-normalizable generalized eigenvectors 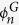 of 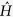 with complex *E*_*n*_ with *n* = 0, 1, 2,...,


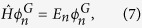


GVs furnish a generalized basis for a normalizable wavepacket, so that


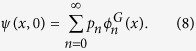


In (8) *ψ*(*x*, 0) is the initial state, the discrete part is *not* the usual spectrum of normalizable bound states with real valued eigenvalues (which is not present here), but an ensemble of vectors with complex eigenvalues *E*_*n*_ = −*i*Γ_*n*_/2 with Γ_*n*_ corresponding to the decay rates^8^,^17^,^19^,^20^. In (8) *p*_*n*_ are the projection of the initial state on the GVs. In the case of the RO, the GVs profile can be expressed as analytical prolongations in the complex plane of the eigenfunctions of the quantum HO^17^.

We have


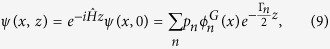


which shows that the evolution of each of the GVs is simply an exponential decay. For the RO the decay rates are quantized, i.e.,


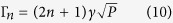


The wavefunction upon evolution can be hence written as a superposition of functions that decay exponentially in the *Z* direction. The components of this expansion can be calculated analytically or numerically, and the resulting scenario predicts quantized decay constants Γ_*n*_^17^. We remark that this analysis is valid in the regime for the generation of photothermal shock waves, and we show in the following that it provides a comprehensive description of experimental results. We also address how the exponent Γ_*n*_ scales as the square root of the beam power, which comfirms the nonlinear origin of the process, as the standard linear decay rates do not depend on the beam power. The power dependence of Γ_*n*_ is an experimentally testable prediction, which directly allows to discriminate GVs from other damping mechanisms, as scattering or absorption.

## Experimental realization of the reversed oscillator

Following the previous theoretical arguments we realize a physical system described by a RO. We generate a quantized reversed oscillator in a nonlocal defocusing photothermal system^21^, as detailed in methods. Nonlinearity has a fundamental advantage: the observed decay rates, Γ_*n*_, are intensity-dependent so that they can be easily distinguished from loss, which is not included in the RO Hamiltonian.

**Figure 2:**
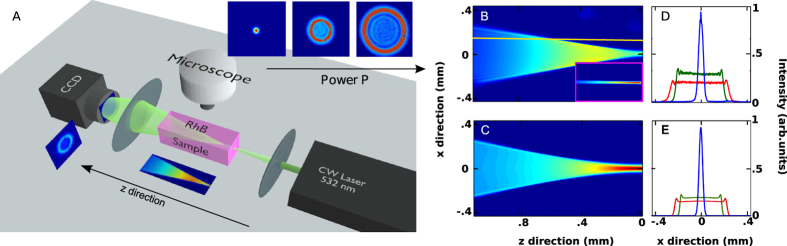
Laser light simulating a quantum reversed oscillator. (**A**) Schematics of the experimental setup to obtain transmitted and top fluorescence images of the laser beam propagating in the RhB samples. The input lens focuses the laser beam into the sample. The collecting lens images the exit plane of the sample onto the CCD camera. The microscope placed above the sample captures top-view images of the laser beam along the propagation direction. The insets show the transverse intensity distribution at the output of the sample for three increasing laser powers, *P*. (**B**) Top-view fluorescence image, for a ≃10 *μ*m waist at *P* = 400 mW. The excitation of the nonlinear response of the sample is signaled by the strongly diverging funnel shape of the beam. The inset shows the z-propagation of the beam as occurs at the lower power, *P* = 10 mW. (**C**). Numerical simulations for panel B. (**D**,**E**) Respectively experimental (**D**) and numerical (**E**) sections of the top-view images taken at *z* = 0.2, 0.6 and 0.9 mm.

In Fig. 2A we illustrate the experimental setup (see Methods) and the top-view imaging apparatus. Figure 2B reports the observed laser beam propagation and Fig. 2C the numerical calculation from the propagation equation (3).

At high power, following previously reported experiments^12^ a shock wave generation is observed and the beam displays a strongly divergent funnel shape, the signature of optical nonlinearity in the RhB solution. This regime is characterized by a fast and power dependent beam decay along the propagation *Z* direction.

At low powers, beam propagation does not manifest the strong divergence and is dominated by diffraction (see inset of Fig. 2B).

The comparison between the experimental and numerical features of the RO eigenstates are reported in Fig. 2D,E, where three transverse sections of the intensity profile at different propagation distances *z* = 0.2, 0.6 and 0.9 mm are displayed.

**Figure 3:**
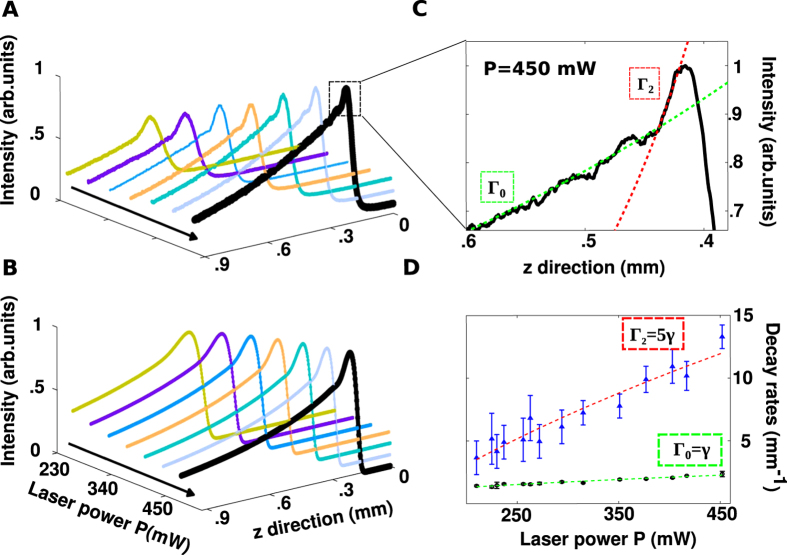
Quantized decay rates. (**A**) Observed intensity decay at different laser powers as obtained by slicing along the propagation z-direction the top-view intensity distribution (see the yellow line in Fig. 2E). (**B**) Numerically calculated decays in the conditions of panel (**A**). (**C)** Enlargement of the peak region of the experimental curve at *P* = 450 mW. The double exponential decay unveils the existence of two exploding states, the fundamental state, n = 0 (slow decay) and the excited state, n = 2 (fast decay). (**D**) Decay rates vs *P* for the fundamental state, Γ_0_ (filled circles) and the excited state, Γ_2_ (triangles).

To demonstrate the excitation of GVs, we show that beam spreading is well described by a superposition of power dependent exponential functions. This leads to the observation of quantized decay rates reported in Fig. 3. The decay dynamics at different power levels, shown in Fig. 3A,B, are obtained slicing the intensity profile along the propagation *z*-direction (see yellow line in Fig. 2E). The signature double exponential behavior is most evident at high power (Fig. 3C).

Observed and calculated double-exponential decay dynamics are found to obey the quantized spectrum scaling Γ_2_/Γ_0_ = 5 at all investigated power levels, *P* (Fig. 3D).

This demonstrates that we excite the fundamental state (n = 0) with decay rate Γ_0_ and the second excited state (n = 2) with Γ_2_ = 5Γ_0_. The state *n* = 1 is not excited, as expected from the input beam symmetry. Each of the two rates is found to have a square root dependence on *P* (see the superimposed dashed lines in Fig. 3D), the signature of the underlying nonlinearity. This power dependence distinguishes RO dynamics from linear loss, due to absorption and scattering.

## Discussion

Wavefunction collapse and irreversibility are basic examples of commonly observed phenomena that cannot be deduced from dynamical equations. The standard description of these effects invokes the artificial interaction with macroscopic detection systems and heat baths. Attempts to circumvent this scheme rely on the introduction of quantized reversed oscillators, as originally discussed by Glauber in measurement theory and by A. Bohm and others for irreversible quantum mechanics. The fundamental innovation of this approach is the ability to include the arrow of time as a product of intrinsic dynamical processes, without resorting to an artificial coupling with the environment.

Here we report a first physical realization of a quantized reversed oscillator with its signature discrete decay rates in nonlinear optical propagation. We hence provide an experimental evidence to ideas so far considered only at a theoretical level. Our results demonstrate the excitation of Gamow vectors during shock wave generation, and furnish a novel perspective to the problem of wave-breaking, which can be hence considered as a dynamically irreversible process.

In addition, the occurrence of nonlinear waves sustaining decay and amplification is expected to be relevant for a number of other extreme nonlinear processes, as supercontinuum generation, rogue waves, amplifying material and light propagation in highly nonlinear optical fibers. The widespread occurrence of shock and wave-breaking phenomena in modern physics shows that nonlinear Gamow vectors can be applied in many different frameworks.

Furthermore, ideas from irreversible dynamics may potentially open new roads for designing devices, as lasers and amplifiers, or for the generating coherent white-light, or very short wavelenghts, as extreme ultraviolet or soft X-rays. Finally, the extension of our results to the single-photon and quantum regime may encompass a variety of novel developments, and also furnish additional insights for innovative tests to fundamental physical theories.

## Methods

### Optical nonlinearity

Heat diffusion in the photothermal liquid causes the light-induced perturbation to extend far beyond the beam intensity profile^18^,^22^,^23^,^24^. Light experiences a refractive index with an inverted parabolic spatial distribution.

### Materials

Samples are prepared by dispersing 0.1 mM of Rhodamine B in water. The measured defocusing Kerr coefficient is |*n*_2_| = 2 × 10^−12^ m^2^W^−1^ and the absorption length L_*abs*_ ≃ 1.6 mm at a wavelength of 532 nm^25^.

### Measurements

High resolution transverse intensity distribution measurements are carried out having the beam propagate vertically through the sample, reducing convection in the water. Decay rates are detected by slicing the intensity profile *I*(*x*, *z*) at *x* ≃ 0.1 mm and fitting the intensity versus *z* data with two exponential functions. The analysis was repeated for different samples and at different power levels.

## Additional Information

**How to cite this article**: Gentilini, S. *et al.* Physical realization of the Glauber quantum oscillator. *Sci. Rep.* **5**, 15816; doi: 10.1038/srep15816 (2015).

## Figures and Tables

**Figure 1:**
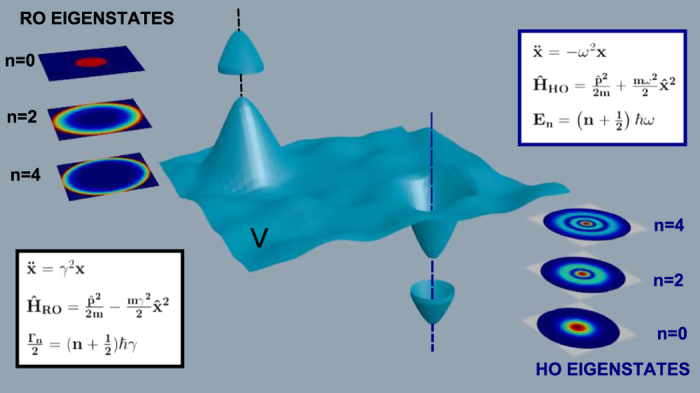
Harmonic versus Reversed Oscillators. Arbitrary potential energy landscape of a system. When the system is in proximity of a local maximum it obeys the Hamiltonian of a RO, 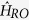. In proximity of the minimum the system obeys the Hamiltonian of a HO, 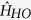. Both the reversed and the harmonic oscillators can occur for a given system as it explores its local energy landscape. The corresponding classical equations for the two Hamiltonians are also indicated in the text boxes. On the left side are reported the exploding eigenstates corresponding to the imaginary part, Γ_*n*_/2, of the eigenvalues of the RO Hamiltonian, 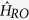. On the right side are shown the bounded eigenstates of the HO associated with increasing quantized energy levels, *E*_*n*_, corresponding to the eigenvalues of the Hamiltonian 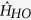.
